# LucY: A Versatile New Fluorescent Reporter Protein

**DOI:** 10.1371/journal.pone.0124272

**Published:** 2015-04-23

**Authors:** Michele E. Auldridge, Hongnan Cao, Saurabh Sen, Laura P. Franz, Craig A. Bingman, Ragothaman M. Yennamalli, George N. Phillips, David Mead, Eric J. Steinmetz

**Affiliations:** 1 Lucigen Corp., Middleton, WI, United States of America; 2 Department of Biochemistry and Cell Biology, Rice University, Houston, Texas, United States of America; 3 Department of Biochemistry, University of Wisconsin, Madison, WI, United States of America; Université de Montréal, CANADA

## Abstract

We report on the discovery, isolation, and use of a novel yellow fluorescent protein. Lucigen Yellow (LucY) binds one FAD molecule within its core, thus shielding it from water and maintaining its structure so that fluorescence is 10-fold higher than freely soluble FAD. LucY displays excitation and emission spectra characteristic of FAD, with 3 excitation peaks at 276nm, 377nm, and 460nm and a single emission peak at 530nm. These excitation and emission maxima provide the large Stokes shift beneficial to fluorescence experimentation. LucY belongs to the MurB family of UDP-N-acetylenolpyruvylglucosamine reductases. The high resolution crystal structure shows that in contrast to other structurally resolved MurB enzymes, LucY does not contain a potentially quenching aromatic residue near the FAD isoalloxazine ring, which may explain its increased fluorescence over related proteins. Using *E*. *coli* as a system in which to develop LucY as a reporter, we show that it is amenable to circular permutation and use as a reporter of protein-protein interaction. Fragmentation between its distinct domains renders LucY non-fluorescent, but fluorescence can be partially restored by fusion of the fragments to interacting protein domains. Thus, LucY may find application in Protein-fragment Complementation Assays for evaluating protein-protein interactions.

## Introduction

Fluorescent proteins (FPs) make it possible to visualize biological processes through *in vivo* imaging and *in vitro* fluorescence labeling. Processes such as protein expression, localization, degradation, and interaction can be observed through fusion of a protein of interest with a FP. Green Fluorescent Protein (GFP) from *Aequorea victoria* and its varied derivatives constitute a multi-colored toolbox ranging from blue to yellow, with red-shifted FPs (RFPs) originating mostly from the sea anemone *Discosoma striata* [1]. The discovery and subsequent explosion in diversity of available FPs and bioluminescent proteins facilitated techniques utilizing Fluorescence or Fӧrster Resonance Energy Transfer (FRET) [2], Bioluminescence Resonance Energy Transfer (BRET), and a specific form of Protein-fragment Complementation Assay (PCA), sometimes called Bimolecular Fluorescence Complementation (BiFC) [3].

In GFP-like and RFP-like fluorophores, fluorescence emanates from a chromophore developed by the formation of an imidazolinone ring system between three centrally located amino acids. Complete maturation of the chromophore necessitates oxidation, making molecular oxygen a strict requirement for these systems [4]. The oxygen- and time-dependence of fluorophore formation, and its irreversibility once formed, limit some applications of these proteins.

We have discovered, cloned, and characterized a fluorescent flavoprotein from a *Geobacillus* species that is homologous to other bacterial MurB enzymes. Members of this family contain a noncovalently bound flavin adenine dinucleotide (FAD) cofactor, and in some cases exhibit bright yellow fluorescence. Although free FAD fluoresces rather weakly (Φ_F_ = 0.032) due to the quenching effects of the adenine moiety, sequestration within the MurB protein environment can enhance its fluorescence [5]. In the case of the *Geobacillus*-derived MurB homologue, FAD fluorescence is enhanced more than 10-fold, making this protein useful as a fluorescent fusion partner.

We have named the novel MurB homologue LucY, for Lucigen Yellow. We observed LucY fluorescence in bacterial and mammalian cells, potentially enabling its use as a fusion partner for applications such as protein localization and for visualization of protein expression and solubility. Furthermore, we have developed a LucY PCA system by which protein-protein interactions can be visualized. Because LucY fluorescence is due to non-covalent binding of FAD, fluorescence development is rapid, distinguishing it from the GFP family. Additionally, the potential reversibility of this fluorescence may enable the use of LucY as a real-time sensor of protein-protein interaction in bimolecular fluorescence complementation.

## Results

### LucY discovery and isolation

LucY was discovered while screening for carbohydrate active enzymes in a metagenomic library prepared from corn stalks using a fluorescent substrate. Microbial cells were cultured from corn stalks from Maple Vane Dairy Farm (Latitude: 43.14116 | Longitude: -89.492702). The field collections were carried out on private land (contact person: John Wagner) and did not involve endangered or protected species. A metagenomic library was prepared, and plates containing several hundred colonies each were assayed for fluorescence using a 365 nm long-wavelength UV lamp. Plasmid DNA was isolated from one colony that exhibited yellow fluorescence. The cloned DNA sequence was compared to the Genbank database using BLASTN [6], and close homology (83% identity) was found to a segment of the *Bacillus licheniformis* ATCC 14580 genome. The cloned region contained homology to bacterial *murG* and *murB* genes and portions of the *spoVE* and *divIB* genes. In other microbes the *murB* gene encodes UDP-N-acetylenolpyruvylglucosamine reductase, a flavoprotein that functions in the synthesis of the peptidoglycan cell wall [7]. The novel *murB* gene encodes a protein with 95% amino acid identity to the uncharacterized *Bacillus licheniformis* MurB protein. A lower level of similarity to the well-studied MurB proteins from *Staphylococcus aureus* (39% identity; 63% similarity) and *Escherichia coli* (26% identity; 43% similarity) was noted. The purified MurB protein from *E*. *coli* exhibits a yellow color [8], and quenching of the fluorescence of the tightly bound FAD cofactor is used to assay binding of compounds to *S*. *aureus* MurB protein [9], suggesting that fluorescence of the metagenomic clone is due to expression of the *mur*B gene.

To confirm that the novel MurB protein is responsible for the observed fluorescence, we cloned its coding region into a bacterial expression plasmid. *E*. *coli* BL21(DE3) cells exhibited bright yellow fluorescence when induced to express the cloned *mu*rB gene. Accordingly, we have adopted the name LucY (Lucigen Yellow) for the novel MurB homologue.

### Purification and fluorescence

**Fig 1:**
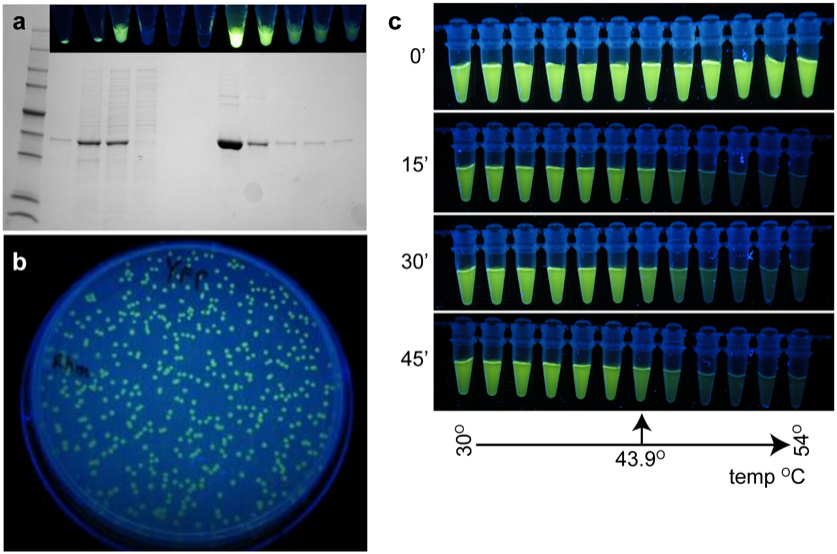
LucY characteristics. (a) His tagged LucY was purified by metal affinity chromatography. Lanes are: protein marker, whole cells, pellet, supernatant, column flowthrough, wash, and elutions. Fractions were photographed under UV light (inset) and are aligned with corresponding gel lanes (b) LucY fluorescence is visible in E. coli colonies grown on a LB plate. (c) Purified LucY was subjected to a temperature gradient (30 to 54°C) in a thermal cycler. Samples were photographed after exposure to elevated temperatures for the time indicated.

LucY is a soluble protein of approximately 33.2kDa and is easily expressed and purified in *E*. *coli* with its fluorescence evident throughout the purification procedure without the addition of exogenous FAD (Fig 1a and 1b). Thus, FAD appears to be tightly bound, as reported for other MurB homologues [8,10]. LucY fluorescence is also evident in mammalian cells (data not shown), which may prove useful for protein localization studies. The fluorescence of purified LucY is moderately thermostable *in vitro* with visible fluorescence remaining for 45 min at approximately 44°C (Fig 1c). Temperatures exceeding 44°C may result in protein instability and therefore release of FAD, suggesting that isolation within the protein increases the intrinsic fluorescence of FAD.

**Fig 2:**
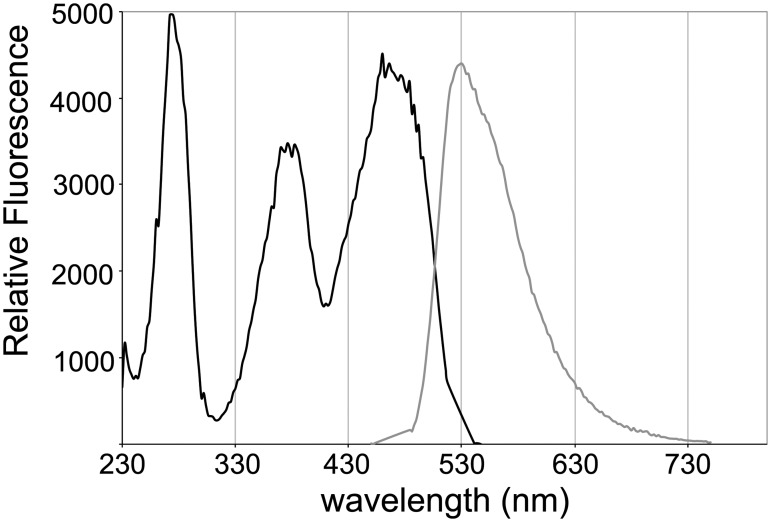
Excitation/emission of LucY. Excitation scan (black) performed with emission at 528nm and emission scan (gray) was performed with excitation at 465nm.

Excitation and emission spectra for LucY are typical for a flavoprotein. Excitation maxima occur at three wavelengths, 276nm, 377nm, and 460nm, and a single emission peak occurs at 530nm (Fig 2). Quantum yield estimates for LucY and other MurB family members were ascertained via the comparative method [11] in relation to the well characterized FAD and flavin mononucleotide (FMN) cofactors. The quantum yield (Φ_F_) of LucY was determined to be on average 0.354, using FAD and FMN as references. Brightness values of the MurB proteins were measured by taking the product of the extinction coefficient and quantum yield for each fluorophore; the values are expressed relative to the brightness of LucY (Table 1). LucY enhances FAD brightness by approximately 10-fold, and it is the brightest of the MurB homologs studied here.

**Table 1:** Fluorescence characteristics of LucY and other MurB homologs.

	Excitation Max (nm)	Emission Max (nm)	Ɛ (M^-1^cm^-1^)	Φ_F_ based on FAD	Φ_F_ based on FMN	Brightness (%of LucY)
FAD	270/375/452	522	11,900	-	0.032	9
FMN	268/376/448	525	12,500	0.273	-	82
**LucY**	**276/377/460**	**528**	**11,662**	**0.356**	**0.351**	**100**
*S*. *aureus*	271/377/460	527	13,511	0.224	0.221	73
*E*. *coli*	276/369/460	527	12,008	0.109	0.108	32
*T*. *thermophilus*	276/375/460	522	13,745	0.051	0.051	17

Ɛ, extinction coefficient

Φ_F_, quantum yield

Published Φ_F_ for FAD and FMN are 0.032 and 0.27, respectively.

### Three-dimensional structure of LucY

**Fig 3:**
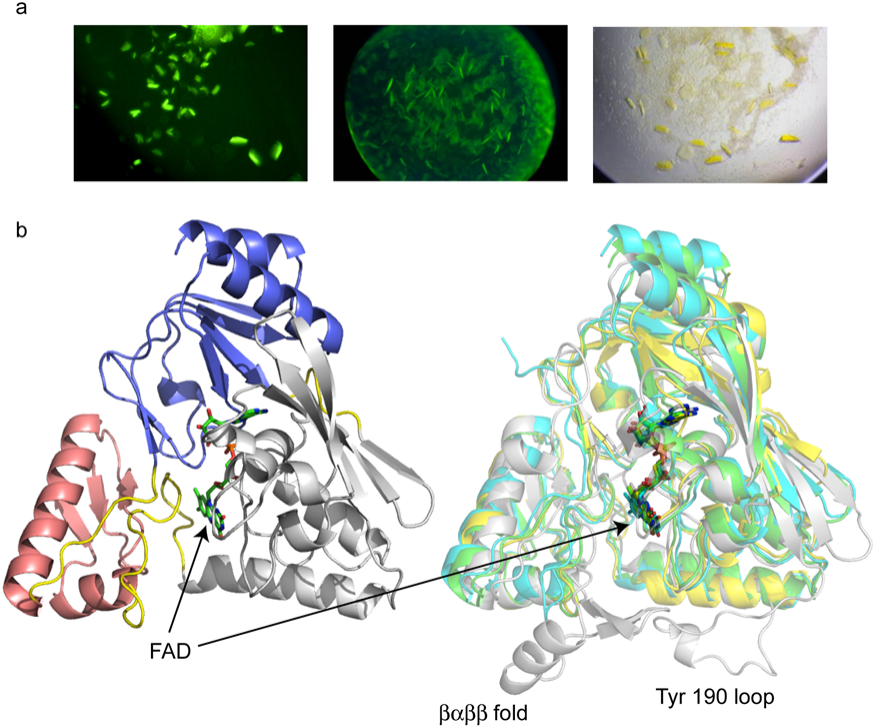
Crystal structure of LucY. (a) LucY crystal trials as seen under UV light (left and middle panel) and visible light (right panel). (b) Left, three-domain fold of LucY. Domains 1 (blue), 2 (white), 3 (pink), and linker loops (yellow) are shown. Right, structure alignment of MurB enzymes: LucY (green), S. aureus (cyan), E. coli (white), and T. caldophilus (yellow). LucY is a type I MurB which lacks the βαββ fold and Tyr 190 loop found in E. coli. FAD cofactors are shown as sticks, nitrogen in blue, oxygen red, and phosphorus orange.

The high-resolution structure of LucY comprises 302 amino acid residues and displays a three-domain fold in complex with the FAD cofactor, similar to the previously published structures of related MurB enzymes [10,12,13,14,15,16] (Fig 3). Domain 1 (N-terminal residues 1–83 and C-terminal 297–302) consists of two α-helices and six β-strands forming a four-stranded mixed β-sheet and a two-stranded parallel β-sheet. Domain 2 (residues 90 to 215) is formed by a five-stranded antiparallel β-sheet and three α-helices. Domain 3 (residues 235–288) contains a three-stranded antiparallel β-sheet and two α-helices. The binding-pocket of FAD is formed primarily by domains 1 and 2, with additional contributions from domain 3 and the loop connecting domains 2 and 3. Domains 2 and 3 potentially form the substrate binding pocket, based on the structures of substrate-bound MurB of *E*. *coli* and *T*. *caldophilus* [13,15]. However, we did not attempt to capture the substrate within the crystal, nor did serendipitous binding of the substrate occur.

LucY shows overall structural similarity to other enzymes in the Type II MurB family from bacteria, including *Listeria monocytogenes* [14], *Staphylococcus aureus* [10], and *Thermus caldophilus* [15]. Structural differences from the *E*. *coli* [12,13] and *Vibrio cholerae* [16] Type I MurB enzymes include the absence of the previously named Tyr190 loop in Domain 2 and lack of the single split *βαββ* fold in Domain 3. Atomic positional root mean squared deviation (RMSD) values of LucY in comparison to the reported free form of several MurB structures are 1.29 Å (*L*. *monocytogenes*), 1.33 Å (*S*. *aureus*), 1.87 Å (*T*. *caldophilus*), and 1.64 Å (*E*. *coli*), based on structural alignment by the PDBefold server [17]. This deviation is generally consistent with the level of sequence identity to LucY of 43.1%, 41.1%, 39.5%, and 30.2%, respectively. The *T*. *caldophilus* enzyme is the exception for which the largest RMSD is observed among the four structures. Interestingly, we found the substrate-bound forms of *T*. *caldophilus* enzyme (PDB code: 2GQU, RMSD 1.45 Å) *and E*. *coli* enzyme (PDB code: 2MBR, RMSD: 1.51 Å) in complex with UDP-*N*-acetylenolpyruvylglucosamine have smaller RMSD values to LucY than the corresponding free forms. This observation suggests LucY might require less conformational change to bind the substrate than do the *T*. *caldophilus* and *E*. *coli* homologues, or that a conformation similar to the substrate-bound complex occurs in the LucY crystal structure.

One copy of the enzyme was observed per asymmetric unit, and the structure belongs to the trigonal space group R32. Interestingly, three crystallographic symmetry-related protein molecules formed an apparent fifteen-stranded β-barrel-like superstructure (S1 Fig). However, the interface area between each LucY molecule forming the β-barrel was calculated to be too small (445.4 Å^2^ via PISA server [18]) for physiologically relevant trimerization. No such crystallographic symmetry-related β-barrel interfaces were formed for other available MurB enzyme structures. In addition, *E*. *coli* MurB was reported to be monomeric in solution [8]. Moreover, an alternative crystal form was found for LucY and resolved to 2.0 Å. This crystal belongs to the C2221 space group and does not contain the pseudo-trimer. Monomers of each form align well with an RMSD of 1.2 Å (over 293 residues, where residues 210–218 are disordered, data not shown).

Ultracentrifugation sedimentation analysis confirmed the presence of a dominant monomeric species, independent of the presence of Mg^2+^ (S2 Fig). Thus, we conclude that the fifteen-stranded β-barrel is a feature of the crystal packing instead of a true trimerization interface. Clear electron density was observed for residues 1–302, FAD, and three hexahydrated magnesium ions, but not for Arg 303 or the C-terminal hexa-His tag. All three magnesium ions in the structure are bound at the surface of the protein and appear to mediate crystal packing between symmetry-related molecules via interaction with the carboxylic groups of Asp 42 and Glu 286, amino groups of Lys 41 and backbone carbonyl groups of Lys 41, Glu 286, and Lys 287. Two of the magnesium ions have occupancies of 0.30 and 0.29 and are located at the three-fold crystallographic symmetry axis making their total occupancy 0.85 and 0.9, respectively.

### The LucY PCA system for protein-protein interaction studies

The reassembly of an artificially split monomeric protein was first demonstrated with ubiquitin [19]. This concept has since been exploited for use with reporter proteins such as GFP [20] and luciferase [21]. In practice, a reporter protein is split into fragments that do not assemble spontaneously, and each fragment is expressed as a translational fusion to one of a pair of proteins of interest for which protein-protein interaction is a possibility. If the two fusion partners interact, the activity of the reporter protein is restored, and an output signal such as fluorescence is generated.

**Fig 4:**
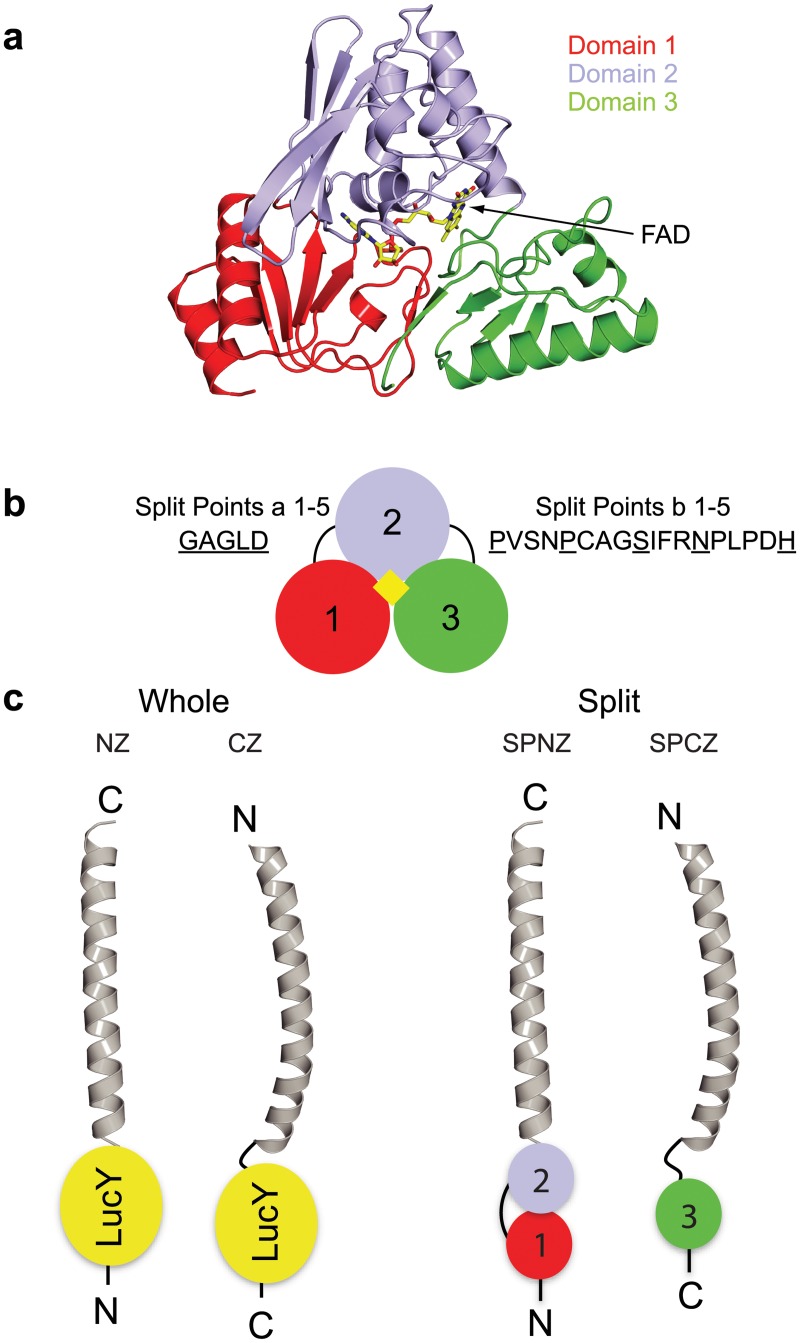
LucY PCA System. (a) LucY is divided into 3 distinct domains (colored red, blue, and green) with (b) a five residue loop connecting domains 1 and 2 and an approximately 18 residue loop connecting domains 2 and 3. Five points within each of these loops were chosen as Split Points (SP) (underlined). (c) Synthetic antiparallel leucine zippers (gray) were used as protein interaction models to test reconstitution of LucY PCA fragments (right) and were compared to whole LucY fusions (left). LucY fused at the N-terminus of the leucine zipper is referred to as NZ, and LucY fused at the C-terminus is CZ.

LucY comprises three discrete domains (Figs 3 and 4a), suggesting candidate split points may lie between domains. Five locations were tested for fragmentation points within the short loop between domains 1 and 2 (residues 84 to 88) and within the long loop between domains 2 and 3 (residues 215–235) (Fig 4b). Split Points were designated “SPa 1–5” and “SPb 1–5”, respectively. LucY N- and C-terminal fragments were fused to synthetic, idealized leucine zippers as a model for interacting protein partners [20]. Because these leucine zippers interact in an antiparallel fashion, one fragment of LucY is fused at the amino-terminus of a leucine zipper, and the complementary LucY fragment is fused to the carboxyl-terminus of the partner leucine zipper. These fusions are designated Split Points NZ 1–5 and CZ 1–5, respectively (Fig 4c; and S1 Table). The CZ split point immediately follows the residue at the carboxyl end of the NZ split point. Each fragment pair was tested for protein expression. Their fluorescence when brought together by the zippers was compared to that of NZ and CZ constructs, which contain the leucine zippers fused to fully intact LucY. Fluorescence was determined visually in whole cell pellets and quantitatively with a fluorometer.

**Fig 5:**
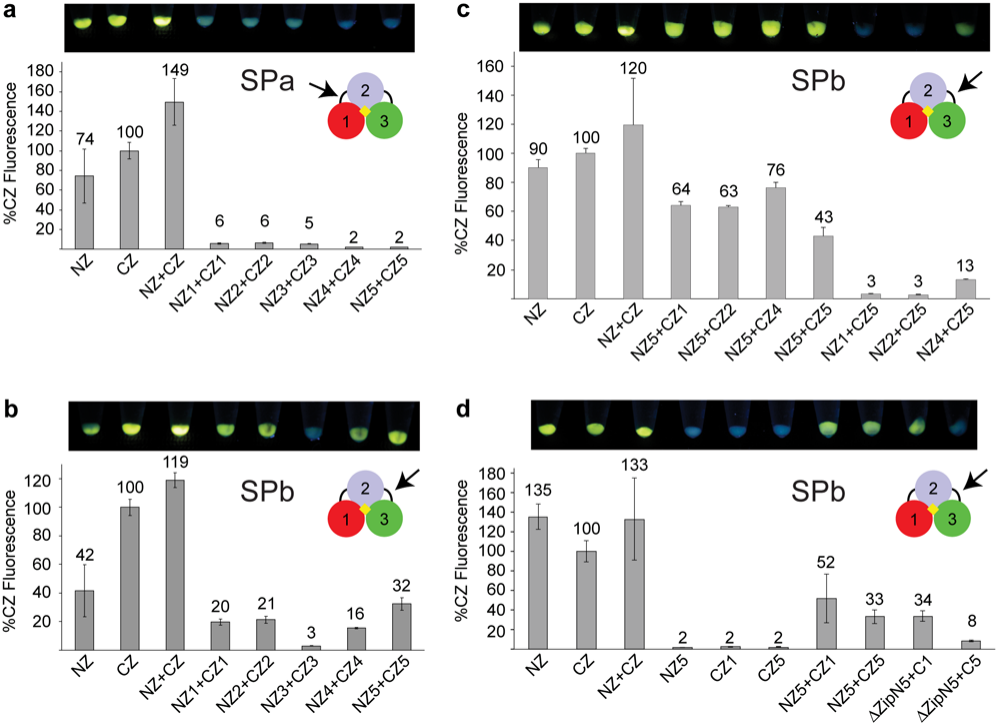
LucY reassembly. (a) Five split points between domains 1 and 2, referred to as SPa NZ or CZ 1–5, were fused with leucine zippers and co-expressed. Image above each graph shows whole cell pellets of corresponding sample under UV light. Fluorescence is represented as a percentage of CZ. Error bars represent standard deviation from the mean (n = 3). (b) Five split points between domains 2 and 3 (SPb NZ or CZ 1–5) were fused with leucine zippers and co-expressed. (c) SPbNZ5 was paired with other split points to determine pair with highest fluorescence. (d) SPbNZ5 paired with either SPbCZ1 or SPbCZ5, was tested for fluorescence independently and without leucine zipper (ΔZip).

Fragmenting LucY at any of the five residues between domains 1 and 2 did not result in fluorescence when the fragments were fused to leucine zipper pairs and co-expressed (Fig 5a). A contributing factor may have been the low expression of the CZ fragment (domain 2 and 3) of each of the split points in comparison to the complementary NZ fragment (domain 1) (S3 Fig). Bright fluorescence was seen in four out of the five pairs split between domains 2 and 3, with highest fluorescence seen with the SPbNZ5 (ending at His 234) and SPbCZ5 partners (beginning at Ala 235) (Fig 5b). All fusions expressed well, and expression levels did not correlate with fluorescence (S3 Fig). Interestingly, the one split point combination between domains 2 and 3 that did not show fluorescence was at Ser 225, a residue previously shown to be important for catalysis [12]. Because none of the splits between domains 1 and 2 could be successfully reconstituted, we chose to focus further analyses on the splits between domains 2 and 3.

Because the reconstituted SPbNZ5 and SPbCZ5 showed the highest fluorescence, we tested each with other split points between domains 2 and 3 to determine if overlaps or gaps in the amino acid sequence would affect fluorescence (Fig 5c). The most successful reconstituted pair was SPbNZ5 with SPbCZ4, which contains a 4 residue overlap of residues 230–234. Interestingly, the opposite pair (SPbNZ4 and SPbCZ5) did not show substantial fluorescence. To verify that neither fragment of LucY was fluorescent on its own and that the leucine zippers were driving the interaction, we tested SPbNZ5 and SPbCZ4 independently and without their leucine zipper fusions. Neither SPbNZ5 nor SPbCZ4 was fluorescent on its own; however, removal of the leucine zipper caused a loss of SPbC4 expression (S4 Fig). Removal of the leucine zipper from SPbNZ5 did not affect expression, but its solubility was improved. When expressed on its own, SPbNZ5 was less soluble than when co-expressed with SPbCZ4 (S4 Fig). Because the zipperless SPbC4 could not be expressed, we returned to the remaining SPbCZ fragments that exhibited fluorescence when co-expressed with SPbNZ5. Only SPbC1 and SPbC5 expressed when their zippers were removed (S4 Fig); therefore, these were chosen for further study. We found that the SPb:NZ5+CZ5 pair possessed the highest level of fluorescence (33% of CZ control) with the least background (8% of CZ control) (Fig 5d).

### Circular permutation of LucY

**Fig 6:**
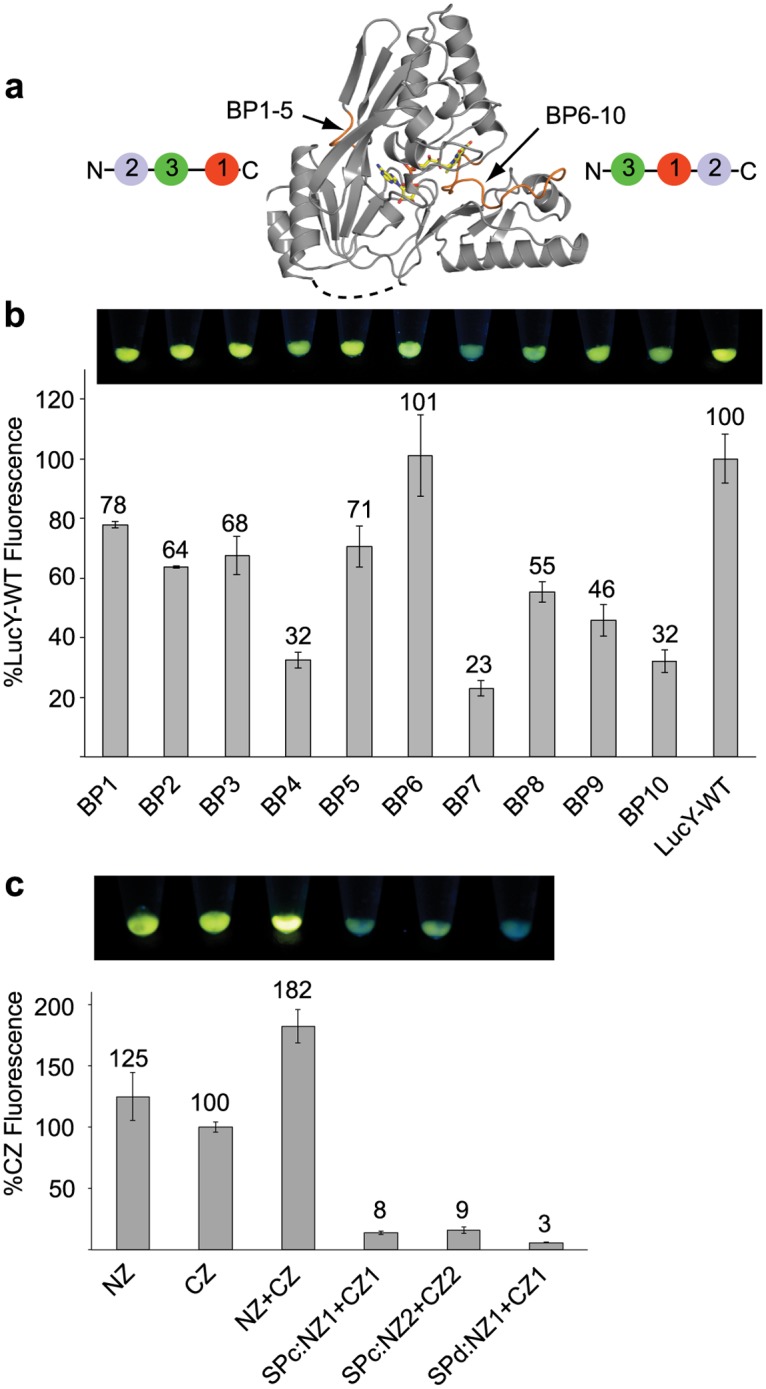
Circular permutation of LucY. (a) Domains 1 and 3 were connected with a small linker (dashed line), and new N and C-termini were created by making Break Points (BP) between domains 1 and 2 (BPs1-5) or between domains 2 and 3 (BPs6-10). (b) Fluorescence of circularly permutated LucY molecules was compared to wild type. (c) Split points were made from a circular permuted LucY such that domains 3 and 1 make up one half and domain 2 makes up the other. Fluorescence of the co-expressed pair is represented as a percentage of CZ. Image above each graph shows whole cell pellets of corresponding sample under UV light. Error bars represent standard deviation from the mean (n = 3).

Reorganization of a polypeptide chain through circular permutation has been used as a means to introduce change into a protein scaffold without amino acid substitutions [22]. It has been successfully investigated with GFP [23]. A welcome consequence to domain reorganization of LucY would be an increase in fluorescence due to increased binding affinity to FAD. Moreover, novel termini offer variation in points of attachment to fusion partners, as well as new possibilities in split points. The wild-type N- and C-termini are 16.7Å apart, requiring a linker greater than four residues. To build circularly permuted LucY, two tandem copies of the gene were joined with a linker encoding 6 amino acid residues. Deletions within this construct were introduced such that new N and C termini were created between either domains 1 and 2 or between domains 2 and 3 (Fig 6a). The same residues that were used as split points above were selected as N- and C-terminal breakpoints (BPs) here (S2 Table). Ten circularly permuted LucY proteins were made: BP1-5 consisted of a 2-3-1 domain arrangement, while BP6-10 consisted of a 3-1-2 domain arrangement.

Circularly permuted LucY proteins all showed some degree of fluorescence (Fig 6b); however, none were any brighter than wild-type. The best candidate was BP6, which has a 3-1-2 domain arrangement, beginning at the long loop that connects domains 2 and 3 in the wild-type protein. Four out of the 10 circular permutation trials resulted in poorly expressed variants (S5 Fig).

New potential split points for PCA became available by reorganizing the domain architecture of LucY, such that domains 3 and 1 could make up one LucY fragment and domain 2 could make up the other. To determine if a LucY PCA system of this type was more useful than previous trials, we made 3 additional split pairs: SPcNZ1/CZ1, SPcNZ2/CZ2, and SPdNZ1/CZ1. SPc pairs consist of domain 2 as the SPNZ fragment and domains 3 and 1 as the SPCZ fragment. SPd consists of the opposing pairing, with domains 3 and 1 as the SPNZ fragment and domain 2 as the SPCZ fragment (S2 Table). These new split pairings did not exhibit fluorescence when reconstituted, despite high levels of expression (Fig 6c and S5 Fig), confirming that a domain 1+2/domain3 pair (as in SPb fusions) offers the best LucY PCA system.

## Discussion

Fluorescence is a common but not universal property of flavoproteins. Depending on the protein environment, the intrinsic fluorescence of flavin cofactors can be either quenched or enhanced [5]. Early studies noted the yellow color of purified *E*. *coli* MurB protein [8,24], but did not make particular note of bright fluorescence. The MurB enzyme family catalyzes a step in biosynthesis of peptidoglycan, a bacterial cell wall component. In a physiological setting, MurB catalyzes the reduction of uridine diphospho-N-acetylglucosamine enolpyruvate (UNAGEP) to UDP-N-acetylmuramic acid via a hydride transfer from NADPH to the substrate through FAD [7]. Fluorescence of MurB-bound FAD has been exploited for kinetic analysis [25] and for the evaluation of potential antibiotics [9,26]. Our discovery of a brightly fluorescent MurB homolog in a metagenomic library prompted our investigation of this protein, which we have called LucY, as a fluorescent probe for a variety of biomedical and biotechnology applications. This report presents the first detailed analysis and comparison of the fluorescence properties of LucY and other MurB homologs.

LucY is the brightest of the MurBs tested. The fluorescence quantum yield is moderate, approximately 25% as bright as wild type GFP. It is easily expressed and purified from *E*. *coli* as a soluble monomeric protein. We have successfully fused LucY to other proteins and found it to be amenable to circular permutation and fragmentation for use as a PCA reporter.

### Flavin-binding fluorescent proteins

The intrinsic yellow fluorescence of flavoproteins is rarely exploited in nature for its optical emission functions, although it has captured the attention of enzymologists ever since “Old Yellow Enzyme” was used to discover flavin mononucleotide (FMN) and the first precise role of a vitamin [27]. One possible example of FMN-containing proteins in bioluminescence is the yellow fluorescent protein encoded by the *lux*Y gene of a bioluminescent bacterium that was originally designated *Vibrio fischeri* strain Y-1 [28,29]. The natural blue bioluminescence of *V*. *fischeri* is exploited in a symbiotic relationship with Bobtail squid, which harvests the bacteria within its light organ for use in a counter-illumination camouflaging mechanism [30]. The *luxY* gene product can function as an antenna protein, converting the blue bioluminescence of the bacteria to yellow. The significance of the wavelength modulation in squid counter-illumination is unclear. Although the *lux*Y gene product has been purified, the temperature sensitivity of this protein limits its utility for biomedical applications [28,29].

On the other hand, flavoproteins are used as photosensors in a variety of systems. FMN-binding photosensory proteins harbor FMN within a conserved “Light Oxygen Voltage” (LOV) domain, and couple blue light detection within the LOV domain to a biological output through an effector domain. Through mutation of a key cysteine residue involved in photocycling, blue-light photoreceptors YtvA from *Bacillus subtilis* [31,32], SB2 from *Pseudomonas putida* [33,34], and Phot2 from *Arabidopsis thaliana* [35] have been developed as FMN-binding fluorescent proteins (FbFPs) [33,36]. A biotechnological limitation of these proteins is the tendency of LOV domains to form dimers [36].

In its wild type form, LucY expresses well, is readily soluble (Fig 1), is monomeric (S2 Fig), does not undergo a photocycle (data not shown), and is moderately fluorescent with a quantum yield of 0.35. Thus further improvements can be focused on increased brightness with little attention needed for the typical solubility and oligomerization issues that plague other FPs. Furthermore, crystallization trials with LucY had a high rate of success, suggesting that fusion to LucY may increase the stability of a target protein and the probability of crystallization. Screening for positive LucY crystals is facilitated by crystal fluorescence evident with UV illumination (Fig 3a). Although no increase in fluorescence was noted, LucY is also amenable to circular permutation (Fig 6). This characteristic increases its usefulness as a fusion partner by providing alternative N- and C-terminal junctions if the traditional N- and C-termini do not result in a well-behaved fusion protein.

### FAD binding and structural basis for MurB fluorescence variation

Although the overall structures of MurB enzymes are very similar (Fig 3), there is a poor correlation between sequence identity and observed fluorescence. Of the MurB enzymes tested, LucY fluorescence has the highest quantum yield (Table 1), followed by the MurB from *S*. *aureus*, *E*. *coli*, and *T*. *thermophilus*, which has 100% identity with the structurally resolved *T*. *caldophilus*. In particular, *T*. *caldophilus*, which like LucY is a Type II MurB, has a much higher overall sequence identity to LucY than *E*. *coli* Type I MurB; however, it exhibits the least fluorescence. This apparent discrepancy is consistent with the higher overall structural similarity of LucY with the *E*. *coli* enzyme (1.64 Å) than with the *T*. *caldophilus* enzyme RMSD (1.87 Å). The increased fluorescence of LucY over closely related proteins is likely due to a combination of factors. Here we suggest that differences in amino acid configurations within the FAD binding pocket, the conspicuous lack of aromatic residues near FAD, and overall decrease in solvent accessibility all contribute to LucY fluorescence.

**Fig 7:**
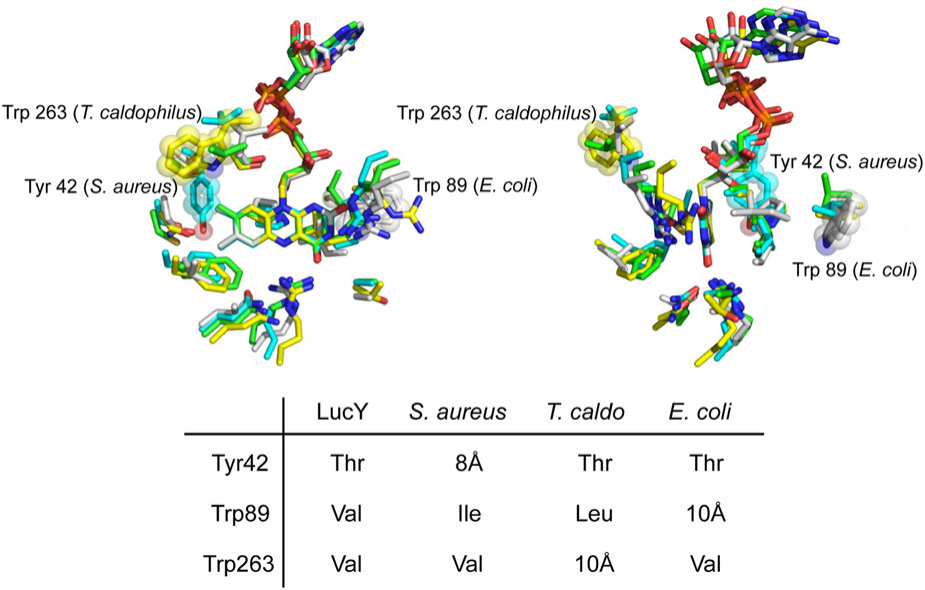
Alignment of active site structure of MurB enzymes. Amino acid residues are aligned within 5 Å of the isoalloxazine moiety of FAD as well as at the positions of the proposed quenching residues Trp 263 (T. caldophilus), Trp 89 (E. coli), and Tyr 42 (S. aureus) shown as spheres and sticks. All other residues and FAD are shown as sticks. Carbon is color coded as follows: LucY (green), E. coli (white), S. aureus (cyan), and T. caldophilus (yellow). Nitrogen is in blue, oxygen red, and phosphorus orange. The right figure is a 90o rotation view of the left figure around the vertical axis. Structure alignment by PyMOL [62] is based on the isoalloxazine moiety of FAD. Inset tabulates the presence of Trp or Tyr in MurBs, with the exception of LucY. Residue numbering corresponds to species with Tyr/Trp. T. caldo for T. caldophilus.

After careful examination of the active site residues directly interacting with FAD, we found residues within hydrogen bonding distance to FAD to be highly conserved (Fig 7). *T*. *caldophilus* enzyme, the least fluorescent of the MurBs tested, shows a different orientation of R151, with its side chain guanidinium group pointing away from flavin in the free form (PDB entry 2GQT), contrary to other MurBs. R151 reorients to come within hydrogen bonding distance to the flavin O2 oxo group upon binding of the substrate UDP-N-acetylenoylpyruvylglucosamine (PDB entry 2GQU) [37]. This may affect the electronic environment of flavin and therefore its fluorescence properties.

Reported mechanistic studies on fluorescence of flavoproteins have shown that fast quenching from the electron-rich aromatic residues, tryptophan and tyrosine but not phenylalanine, can lead to a decrease in the life-time of the excited *S*_*1*_ state of flavin in a manner exponentially dependent on distance. This photoinduced electron-transfer (PET) process forms a Trp^+^/Tyr^+^ and FAD^-^ pair [38,39,40,41,42]. Extremely short center-to-center distances between an electron donor and acceptor of less than 5 Å can lead to strong quenching of fluorescence, with a PET rate on the picosecond time scale. Longer distances of 10 Å contribute to quenching, although at a slower PET rate, usually on the nanosecond scale [39,40,41,42,43,44]. It is also suggested that tryptophan is a stronger quencher likely due to its higher ionization potential [42,44,45].

We hypothesize that PET quenching from the Trp/Tyr residues likely contributes to the fluorescence variation within the MurB family of enzymes in this study. There is a tryptophan approximately 10 Å from FAD in the least fluorescent MurB we studied (Trp 263 in *T*. *caldophilus* MurB). In contrast, the other MurB enzymes have a conserved valine at the position corresponding to *T*. *caldophilus* Trp 263. Moreover, LucY, the brightest of the MurBs, does not contain a Trp or Tyr residue within 10 Å of its FAD binding pocket. Tyr 42 in the second-ranked *S*. *aureus* MurB is at a distance of 8 Å, and Trp 89 in *E*. *coli* lies approximately 10 Å from FAD. No such aromatic residues are observed in LucY. Trp 89 of *E*. *coli* MurB is substituted by Val 105 in LucY, Ile 119 in *S*. *aureus*, and Leu 83 in *T*. *caldophilus*. Tyr 42 in *S*. *aureus* MurB substitutes a conserved Thr in all other MurBs (Fig 7).

Effects from solvent quenching and/or dielectric constant variation in the microenvironment around FAD can affect fluorescence [44,46]. Using CCP4 6.3 areaimol [47,48], we found that *T*. *caldophilus* enzyme has an overall larger solvent accessible area surrounding the flavin moiety (73 Å^2^) compared to its homologues studied above (average among LucY, *S*. *aureus*, and *E*. *coli* is 46.4 Å^2^), which may lead to stronger solvent quenching.

### LucY as a reporter of protein-protein interactions

Cellular systems consist of complex networks of protein-protein interactions. Several methods developed to investigate these interactions rely on genetically encoded fluorescent reporters. Adaptation of fluorescent proteins to a Protein-fragment Complementation Assay enables detection of protein interaction without complicated instrumentation or stoichiometric expression of fragments [49] [50]. Numerous FPs have been developed into reporters of PCA, most notably GFP [20] for bacterial systems and YFP [3,51] for mammalian systems. For a full list of PCA FP reporters, see Kodama et al., [52]. A majority of the FP reporters for PCA are either AvGFP- or DsRFP-derived FPs and thus possess the same limitations regarding slow maturation time and irreversibility [53].

A near-IR split-FP from the engineered *Psuedomonas* bacteriophytochrome iRFP (termed iSplit) has been reported [54]. Despite the benefit of the long wavelength excitation and emission, iSplit also suffers from irreversibility. While this manuscript was in preparation, development of a reversible split PCA from a fluorescent variant of the truncated *Deinococcus radiodurans* bacteriophytochrome called IFP1.4 was reported [55]. Both iRFP and IFP PCA were split within structurally similar loops between domains, so the basis of the difference in reversibility is unclear.

We will continue to explore LucY as a reporter of protein-protein interaction, particularly for use in dynamic studies. Reversible assembly of the complete FAD-binding pocket from split-LucY fragments may provide a rapid on/off fluorescence toggle, potentially enabling a reversible fluorescent reporter for PCA applications. Further advantageous qualities include LucY’s thermotolerance, with fluorescence evident at temperatures as high as 44°C. Fluorescence maturation in GFP-like proteins is typically temperature sensitive [4]. Thermotolerance may prove useful in high temperature experiments in which GFP falls short.

## Materials and Methods

### Cloning

A 10g sample of corn stalks collected from a field outside Middleton, WI in the fall of 2007 was added to YTP-2 medium containing (per liter) 2.0 g yeast extract, 2.0 g tryptone, 2.0 g sodium pyruvate, 1.0 g KCl, 2.0 g KNO_3_, 2.0 g Na_2_HPO_4_^.^7H_2_O, 0.1 g MgSO_4_, 0.03 g CaCl_2_, and 2.0 ml clarified tomato juice. The sample was grown at 55°C and 200 rpm in a 2 L flask containing 1 L of medium. After 2 weeks the media was filtered through Miracloth (EMD Millipore, Philadelphia, PA; pore size: 22–25 μm) and the remaining material was centrifuged to pellet the microbial cells. The cells from the enrichment culture were resuspended in lysis buffer and high molecular weight genomic DNA was purified using the Qiagen (Valencia, CA) Genomic-tip kit. The DNA was randomly fragmented to 3–6 kb using a Hydroshear Apparatus (Digilab, Marlborough, MA), and the ends of the DNA were made blunt using the DNATerminator kit (Lucigen, Middleton, WI). The sheared, end-repaired DNA was gel purified and ligated to the pEZSeq vector (Lucigen, Middleton, WI). The ligation reaction was transformed into E. coli 10G cells (Lucigen, Middleton, WI), the cells were plated on LB media containing 30 μg/ml kanamycin and grown overnight at 37°C. The plates containing several hundred colonies each were moved to a dark room and checked for fluorescence using a 365 nm long wavelength hand held UV lamp (UVP, Upland, CA). The fluorescent colony was grown overnight in terrific broth, the plasmid DNA was purified using standard procedures and the nucleotide sequence of the entire 3684 bp recombinant insert was determined by Sanger sequencing biochemistry on an Applied Biosystems 3100 Genetic Analyzer (Foster City, CA).

### Purification

For bacterial expression and purification, LucY was cloned into the pRham C-His vector (Lucigen). LucY was expressed in 1 liter 0.2% L-Rhamnose auto-induction media overnight at 37°C (http://lucigen.com/Expresso-Rhamnose-Cloning-and-457Protein-Expression-System/). Cultures were harvested by spinning 20 minutes at 5,000 rpm (4,620 Xg) and the pellets flash frozen in liquid nitrogen for storage at -80°C. 7 grams of the cell pellet was lysed via sonication in 35mL 10mM imidazole, 1X PBS pH 8.0. Lysate was clarified by spinning 10,000rpm (12,062 Xg) for 40 minutes. A 5mL bed volume of Ni-NTA resin was equilibrated with 5 column volumes 10mM imidazole, 1X PBS pH 8.0. Clarified lysate was loaded onto the equilibrated Ni-NTA column and then washed with 30 column volumes 10mM imidazole, 1X PBS pH 8.0. LucY was eluted in 5mL fractions with 5 column volumes 500mM imidazole, 1X PBS pH 8.0. Fluorescent elution fractions were pooled and dialyzed overnight in 2 liters 150mM NaCl, 50mM Tris pH 7.5 at 4°C. LucY contained minimal aggregates after dialysis and was spun for 10 minutes at 10,000rpm (12,062 Xg) to clarify prior to storage at -80°C. Final protein concentration was determined to be 8mg/mL using a Bradford assay.

### Spectroscopy

A Tecan Infinite M1000Pro monochromotor-based plate reader (Tecan, Männedorf, Switzerland) was used for fluorescence scans. Excitation/emission scans were run in Greiner FLUOTRAC 200, 96 well, flat-bottom black microplates (Greiner Bio One, Monroe, NC) with emission monitored at 528 nm and excitation at 465 nm. The integral of the emission spectrum was used for quantum yield (ϕ_F_) determinations by comparison to FAD (ϕ_F_ 0.032) or FMN (ϕ_F_ 0.27)[5]. Absorption at the excitation wavelength and integration of the area under the emission spectrum of each sample at five concentrations were measured. Absorbance was plotted against emission integration for each sample and standard, and the slope of a best-fit line for each was determined. The slope of the sample in comparison to the slope and known quantum yield of each standard was used to calculate the quantum yield [11]. Use of FAD and FMN as standards provided an internal control by using FAD to calculate ϕ_F_ for FMN and vice versa and comparing these values with those published.

To determine extinction coefficients (Ɛ) for LucY and MurB homologs, purified protein was denatured by the addition of 6.8M Urea and an absorbance at 450nm was measured. Using Ɛ_450_ = 11,900 M^-1^ cm^-1^ for FAD [56] and the Beers-Lambert Law (Abs = Ɛcl), the concentration of FAD released from denatured protein was calculated. An absorption spectrum of native protein was obtained and their peak absorption was recorded. With this absorption value and the concentration of released FAD, an extinction coefficient for each protein was determined.

Whole cell fluorescence measurements were determined using a BioTek Synergy 2 filter-based fluorometer (Winooski, VT). Emission was measured at 528nm (+/- 20nm) with excitation at 485nm (+/- 20nm). Cells were harvested from a volume such that all samples were normalized to an OD_600_ of 2.0 when resuspended, washed once with PBS and resuspended in 1ml PBS. Three 200μl aliquots per sample were read in 96-well Nunc optical bottom plates (Thermo Scientific, Waltham, MA). The readings are expressed as a percentage of the average CZ readings and then those percentages are averaged and a standard deviation of the mean is calculated for each sample.

### Crystallization, diffraction data acquisition and structure determination

Initial LucY crystallization trials were set-up using microbatch-under-oil and an in-house sparse matrix crystallization screen. The first small scale microbatch-under-oil experiment contained 0.5μl reservoir solution (0.2M MgCl_2_, 0.1M Tris pH 8.5, and 20% PEG 8000) and 0.5μl pure LucY and was overlain with 50μl mineral oil. Crystals were optimized using vapor diffusion by hanging drop by varying the concentrations of PEG and MgCl_2_. LucY crystals of various morphologies formed within 24 hours. Final crystallization conditions contained 1.5 μl reservoir solution (0.5M MgCl_2_, 0.1M Tris pH 8.5 and 20% PEG 8000) and 1.5 μL of 8.0 mg/ml LucY in 150mM NaCl, 50mM Tris pH 7.5. Crystals were cryoprotected by transferring into Mitegen LV CryoOil (MiTeGen, LLC, Ithaca, NY) prior to mounting and were flash-frozen in liquid nitrogen.

Diffraction data were collected at Argonne National Laboratory on the LS-CAT (21-ID-F) beamline using a wavelength of 0.97872 Å and a MAR 225 detector. Data set was collected to a resolution of 1.85 Å, indexed, integrated and scaled using HKL-2000 [57]. The structure of LucY was determined by molecular replacement using AutoMR (Phaser) [58] and Autobuild programs of PHENIX [59] suite with polypeptide coordinates without FAD of *Listeria monocytogenes* MurB as the search model (Protein Data Bank (PDB) entry 3TX1, [14]). One FAD ligand was added to the model with COOT [60] based on the clear electron density in the *F*_*o*_ *-F*_*c*_ omit map. The structure was completed with alternating rounds of manual model building with COOT and refinement with PHENIX. Water was added and updated during refinement. Three hexahydrated magnesium ions [Mg(H_2_O)_**6**_]^2+^ were added to the structure based on density in the *F*_*o*_ *-F*_*c*_ omit map which is consistent with the presence of magnesium ion in the crystal drop.

The final structure was refined to a resolution of 1.85 Å with an *R*_cryst_ of 0.207 and an *R*_free_ of 0.238. Model quality was assessed using MolProbity [61]. The final structure files of were deposited into Protein Data Bank at www.rcsb.org; the PDB entry number of LucY is 4PYT. Data collection and refinement statistics are summarized in Table 2. All structures in the figures have been rendered using PyMOL [62].

**Table 2:** Statistics for data collection and refinement.

Statistic	LucY
Protein Data Bank code	4PYT
Spacegroup	R32
Cell dimensions	
*a*, *b*, *c* (Å)	106.8, 106.8, 201.1
*α*, *β*, *γ* (°) Wavelength (Å)	90.0, 90.0, 120.0 0.9787
Resolution range of data collection (Å)	44.17–1.85 (1.88–1.85)
No. of reflections (measured/unique)	259555/37611
Completeness % (Å) Redundancy	99.8 (98.4) 6.9 (3.8)
*R*_sym_ ^a^ I/^b^ Resolution range in refinement (Å) No. of unique reflections (work/test)	0.092 (0.86) 18 (1.2) 44.17–1.85 37607/1876
*R*_cryst_ ^c^	20.4 (30.1)
*R*_free_ ^d^	22.7 (30.5)
Mean coordinate error, maximum likelihood based (Å)	0.24
Rmsd bond length (Å)	0.007
Rmsd bond angles (°)	1.42
Average B value (Å^2^) (overall/protein/waters/ligands) Real space CC (overall/FAD/isoalloxazine)	36.0/35.2/41.9/29.4 0.914/0.897/0.837
No. of non-hydrogen atoms in refinement No. of protein atoms No. of protein residues No. of waters No. of ligand molecules Ramachandran Statistics^e^ (%)	2698 2294 302 328 1 FAD, 3 Hexahydrated Mg^2+^, 2 Cl^-^ 98.00, 2.00, 0

Values in parenthesis are for the highest resolution shell.

^a^ *R*_sym_ = Σ_*hkl*_ Σ_*i*_ | *I*_*i*_(*hkl*)- 〈*I*(*hkl*)〉| / Σ_*hkl*_ Σ_*i*_ *I*_*i*_(*hkl*), where *I*_*i*_(*hkl*) is the intensity of an individual measurement of the symmetry related reflection and 〈*I*(*hkl*)〉 is the mean intensity of the symmetry related reflections.

^b^I/σ is defined as the ratio of averaged value of the intensity to its standard deviation.

^c^ *R*_cryst_ = Σ_*hkl*_ ||*F*_obs_|—|*F*_calc_||/ Σ_*hkl*_ |*F*_obs_|, where *F*_obs_ and *F*_calc_ are the observed and calculated structure-factor amplitudes.

^d^ *R*_free_ was calculated as *R*_cryst_ using randomly selected 5% of the unique reflections that were omitted from the structure refinement.

^e^Ramachandran statistics indicate the percentage of residues in the most favored, additionally allowed and outlier regions of the Ramachandran diagram as defined by MOLPROBITY.

### Analytical Ultracentrifugation

LucY was subjected to equilibrium ultracentrifugation in a Beckman Coulter XL-A analytical ultracentrifuge (Beckman Coulter, Pasadena, CA). Protein was dialyzed against 50 mM Tris, pH 7.5, 150 mM NaCl with or without 10 mM MgCl_2_. Approximately 100 μL of sample was placed in double-sector charcoal-filled Epon centerpieces with ~110 μL dialysate as reference. Equilibrium data were collected at 4°C for high and low protein concentrations with Mg^2+^ and a single high concentration without Mg^2+^. Equilibrium was ascertained as superimposable gradients collected 2–3 hrs apart. Speed for equilibrium data were 9,200, 13,000, 18,000 and 24,000 rpm. Gradients were monitored at 270 nm and, for a subset, at 380 nm to specifically monitor the flavin contribution. The partial specific volume of the protein was computed from the supplied sequence as 0.742 ml/g; no correction was made for temperature, charge or flavin. The molecular mass, excluding the flavin, was computed as 33,725 Da and extinction coefficient was calculated to be 18,450 M^-1^ cm^-1^.

### LucY PCA construction and development

Constructs containing an idealized leucine zipper pair and full length LucY were made such that in one variation LucY was fused to the N-terminus of a zipper (NZ) and in the other variation LucY was fused to the C-terminus of the complementary zipper (CZ), similar to that described in [20]. NZ consisted of a 6x-His tag, followed by full length LucY, a short linker sequence (GGSGSS) and the sequence encoding for the following idealized leucine zipper ALKKELQANKKELAQLKWELQALKKELAQ. CZ consisted of the sequence encoding for the complementary leucine zipper partner, EQLEKKLQALEKKLAQLEWKNQALEKKLAQ, a short linker sequence, GGSG, LucY, a longer linker sequence (SLSTPPTPSTPPT) and an Avi-tag (Avidity, Aurora, CO). Leucine zippers were incorporated into primers and purified PCR products were inserted into vectors already containing full length LucY by nature of their overlapping ends via recombinational cloning [63,64]. Plasmids containing NZ and CZ were chosen for their compatible origins of replication, differing antibiotic resistances, and promoters that allowed differing induction methods. NZ was placed behind an arabinose inducible promoter into a modified pRanger vector (Lucigen, Middleton, WI) that includes a T7 gene 10 translational leader sequence and we refer to it here as pRex-Ara. CZ was placed into pRham (Lucigen, Middleton, WI) behind a rhamnose inducible promoter.

Split LucY fragments were made from NZ and CZ progenitors by PCR-based deletion. Split point NZ halves terminate at the following residues: SPaNZ1, 2, 3, 4 and 5 at G84, A85, G86, L87, and D88, respectively while SPbNZ1, 2, 3, 4 and 5 terminate at P217, P221, S225, N229, and H234. CZ halves begin at residues immediately following the corresponding NZ half, for example SPaCZ1 begins at A85 (S1 Table).

To assess the efficacy of a split pair, SPNZ and SPCZ fragments were cotransformed into E. cloni 10G chemically competent cells (Lucigen, Middleton, WI). For a typical experiment a sample set included NZ, CZ and cotransformed NZ+CZ as controls. Cultures in 3ml Lauria-Bertani media were grown in the appropriate antibiotic(s) and autoinduced [64,65] using the appropriate sugar inducer(s). Following a 16–18 hour growth at 37°C, cells were harvested and processed for; fluorescence measurement on a Bioteck fluorometer microplate reader, imaging of cell pellets under UV light, and expression analysis on a coomassie stained gel. For each analysis, cells were normalized on the basis of optical density at 600nm.

### Circular Permutation construction and development

The circularly permuted LucY constructs were created first by generating a vector containing a tandemly repeated LucY separated by the linker GGSGSS, TdLucYpRex-Ara. This was created via recombinational cloning by amplifying one copy of LucY from CZpRham, including the linker sequence within the 3’ end and inserting it into NZpRex-Ara. Successful recombinants replaced the zipper sequence with a copy of LucY and the 18bp linker sequence. The new N and C termini, or Break Points (BP), were chosen between domains 1 and 2 and between domains 2 and 3 identical to those chosen for the above described split points. Primers were designed to amplify LucY from TdLucYpRex-Ara such that the break points between domain 1 and 2, BPs1-5, began at residue G84, A85, G86, L87, or D88 and ended at residue L83, G84, A85, G86, or L87. Break points between domains 2 and 3, BPs 6–10, started with P217, P221, S225, N229, or H234 and ended at residue Q216, N220, G224, R228, or D233 (S2 Table). The amplified products were recombinationally cloned into pRham and were therefore rhamnose inducible. From the circularly permuted LucY, we made 6 additional split points (S1 Table, SPc and SPd). These split points were made and their resulting fluorescence and expression were assessed in the same manner as that previously discussed.

## Supporting Information

S1 Fig Hexahydrated magnesium ions mediate crystal packing between symmetry-related LucY molecules. Left, three crystallographically equivalent LucY molecules form an apparent fifteen-stranded β-barrel-like artificial superstructure. Three symmetry-related magnesium ions at the center of the barrel interact with side-chain of carboxylic group of Asp 42, side-chain amino group of Lys 41, and backbone carbonyl group of Lys 41. Right, crystal contact between three LucY molecules mediated by two magnesium ions located coaxial with the 3-fold axis via interactions with side-chain carboxyl group of Glu 286 and backbone carbonyl groups of Glu 286 and Lys 287. FAD and amino acid residues involved in interaction with magnesium ions are shown as sticks. Hexahydrated magnesium ions are shown as non-bonded spheres. Carbon is colored in white, oxygen red, nitrogen blue, phosphorus orange, and magnesium green. *2F*_*o*_ *-F*_*c*_ electron density map is shown at 1σ as blue mesh within 2 Å around the magnesium ions involved in mediating crystal packing. The magnesium ions at the corners of the left figure correspond to the magnesium ions at the center of the right figure.
(PDF)

S2 Fig Plots of absorbance (A270) vs. radial position of highly concentrated LucY with 10 mM MgCl_2_, from low and high speed spins. Raw data is represented by red circles. Fit as a monomer is shown by the red line and as a trimer by the dashed line. The black line shows a fit allowing both monomer and trimer contribution, as well as baseline optical density (BOD) to assess the presence of non-sedimenting absorbance. Results indicate a dominant monomer species. A putative small population of trimer is possible but difficult to quantify, due to small population size and uncertainty in baseline absorption due to free flavin in the solution.
(PDF)

S3 Fig Split Point co-expression. Commassie stained SDS-PAGE gels showing expression of (a) NZ, CZ, and SPa1-5, corresponding to data in Fig 5a. (b) NZ, CZ, and SPb1-5, corresponding to data in Fig 5b. (c) SPbNZ5 and SPbCZ5 paired with non-continuous CZ or NZ partner in whole cells, corresponding to data in Fig 5c. (d) SPbNZ5, SPbCZ1, or SPbCZ 5; alone, in combination, or with their leucine zippers removed in whole cells, corresponding to data in Fig 5d. NZ and CZ bands are indicated by a dot.
(PDF)

S4 Fig Split Point solubility and expression. (a) Commassie stained SDS-PAGE gels showing insoluble (P) and soluble (S) fractions of SPbNZ5 and SPbCZ4, alone or coexpressed with or without leucine zippers. (b) Expression of the C-terminal fragments of LucY without leucine zipper, uninduced (UI) and induced (I). Bands of interest are indicated by a dot.
(PDF)

S5 Fig Expression of circular permutations and splits made from them. Commassie stained SDS-PAGE gels showing expression of (a) each circular permutation, designated BP1-10, and (b) split point trials derived from circularly permuted LucY. Corresponds to data in Fig 6a and 6b.
(PDF)

S1 Table Start and end residues for each split point (SP). NZ constructs start with a 6x-His tag. CZ constructs end with the linker sequence SLSTPPTPSTPPT, followed by an Avi-tag. Split Pairs c and d were constructed from a circular permutation construct.
(DOCX)

S2 Table N- and C-terminal residues of LucY circular permutations, with order of domain occurrence listed. Amino acid numbering is that of wild-type.
(DOCX)
