# Supplementary figures and images for: LucY: A Versatile New Fluorescent Reporter Protein
Source: PLoS One. 2015 Apr 23;10(4):e0124272. doi: 10.1371/journal.pone.0124272 (PMC4408115; doi:10.1371/journal.pone.0124272)

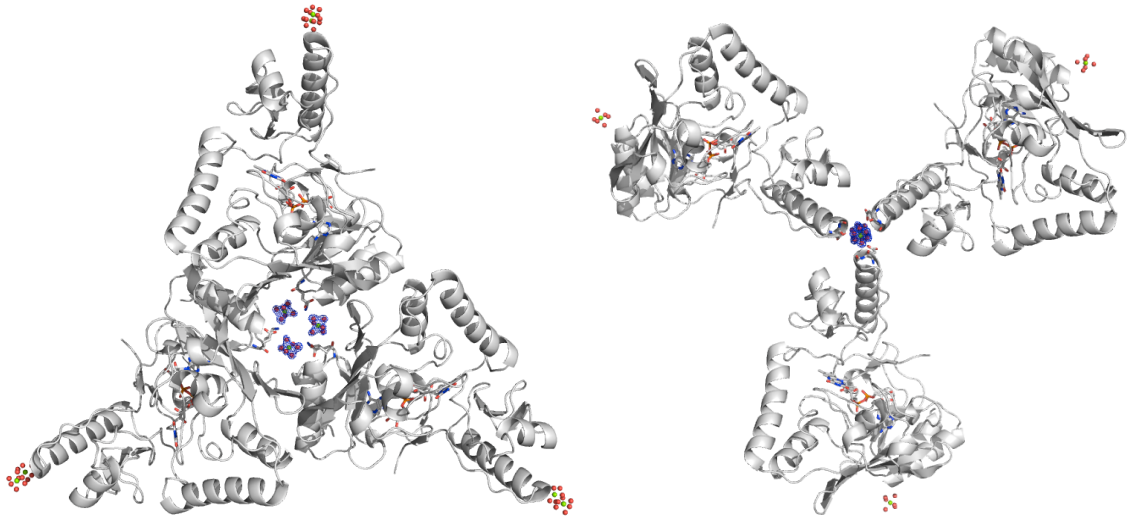

Supplement: S1 Fig — Left, three crystallographically equivalent LucY molecules form an apparent fifteen-stranded β-barrel-like artificial superstructure. Three symmetry-related magnesium ions at the center of the barrel interact with side-chain of carboxylic group of Asp 42, side-chain amino group of Lys 41, and backbone carbonyl group of Lys 41. Right, crystal contact between three LucY molecules mediated by two magnesium ions located coaxial with the 3-fold axis via interactions with side-chain carboxyl group of Glu 286 and backbone carbonyl groups of Glu 286 and Lys 287. FAD and amino acid residues involved in interaction with magnesium ions are shown as sticks. Hexahydrated magnesium ions are shown as non-bonded spheres. Carbon is colored in white, oxygen red, nitrogen blue, phosphorus orange, and magnesium green. 2F o -F c electron density map is shown at 1σ as blue mesh within 2 Å around the magnesium ions involved in mediating crystal packing. The magnesium ions at the corners of the left figure correspond to the magnesium ions at the center of the right figure. (PDF) [file pone.0124272.s001.pdf]

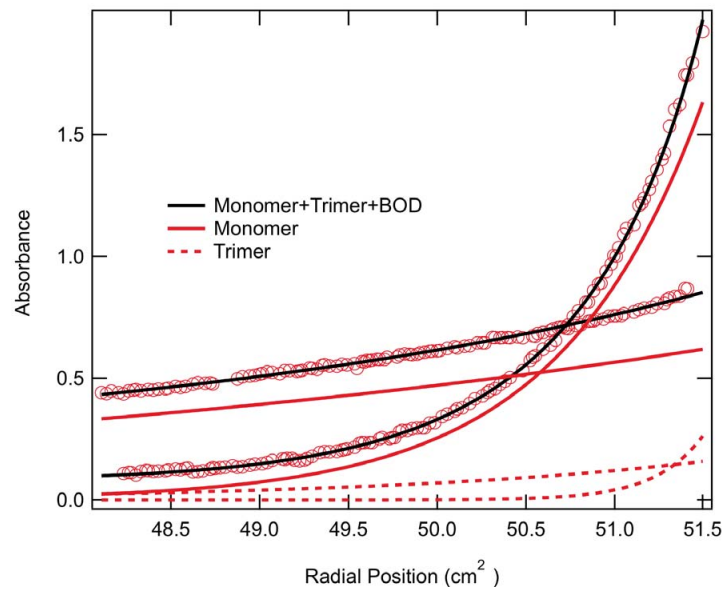

Supplement: S2 Fig — Raw data is represented by red circles. Fit as a monomer is shown by the red line and as a trimer by the dashed line. The black line shows a fit allowing both monomer and trimer contribution, as well as baseline optical density (BOD) to assess the presence of non-sedimenting absorbance. Results indicate a dominant monomer species. A putative small population of trimer is possible but difficult to quantify, due to small population size and uncertainty in baseline absorption due to free flavin in the solution. (PDF) [file pone.0124272.s002.pdf]

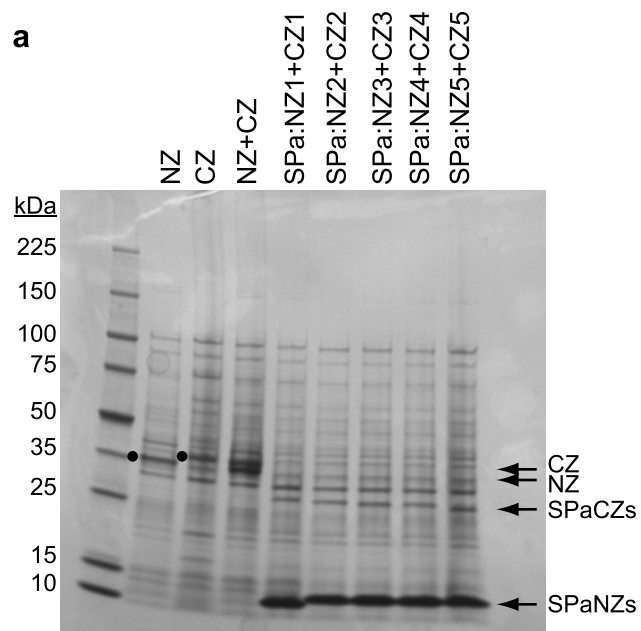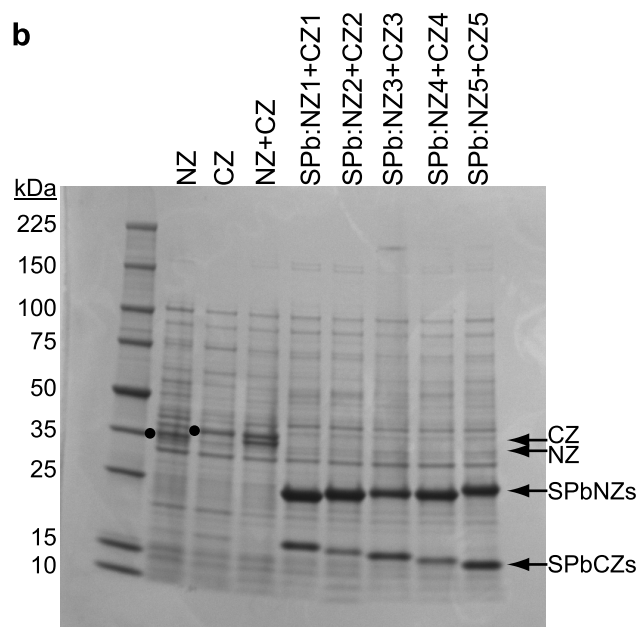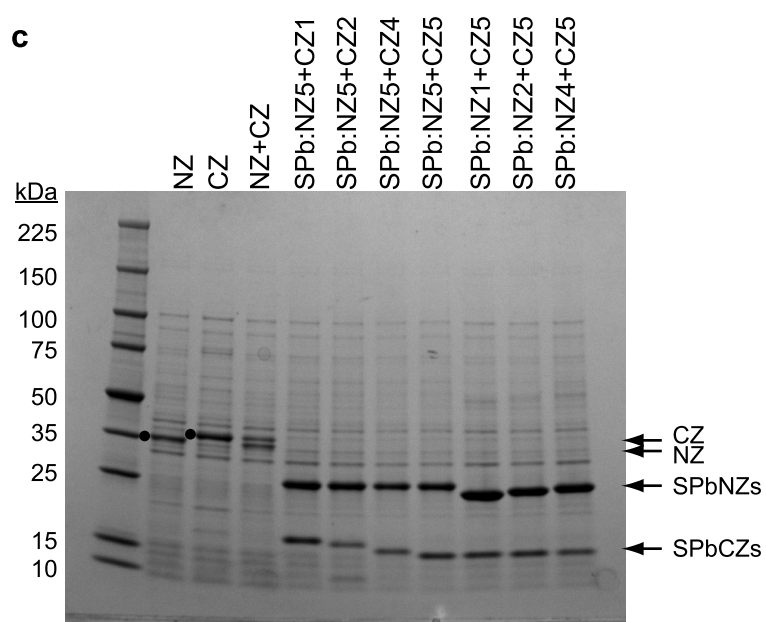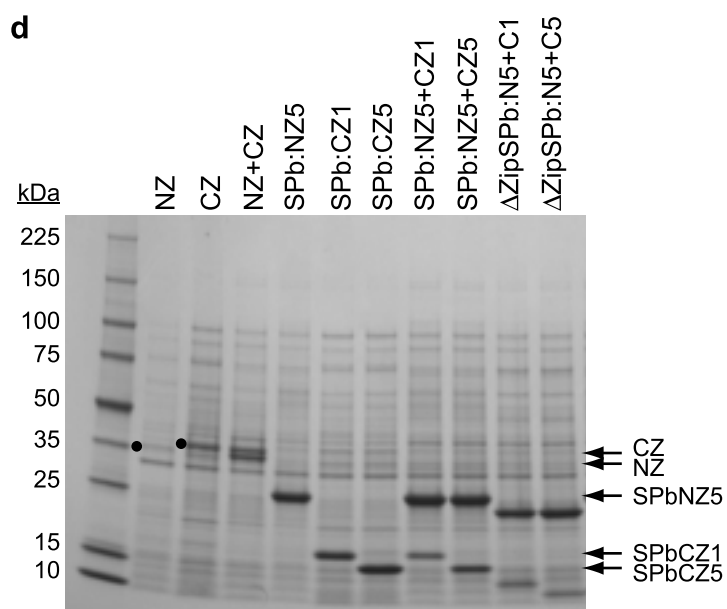

Supplement: S3 Fig — Commassie stained SDS-PAGE gels showing expression of (a) NZ, CZ, and SPa1-5, corresponding to data in Fig 5a. (b) NZ, CZ, and SPb1-5, corresponding to data in Fig 5b. (c) SPbNZ5 and SPbCZ5 paired with non-continuous CZ or NZ partner in whole cells, corresponding to data in Fig 5c. (d) SPbNZ5, SPbCZ1, or SPbCZ 5; alone, in combination, or with their leucine zippers removed in whole cells, corresponding to data in Fig 5d. NZ and CZ bands are indicated by a dot. (PDF) [file pone.0124272.s003.pdf]

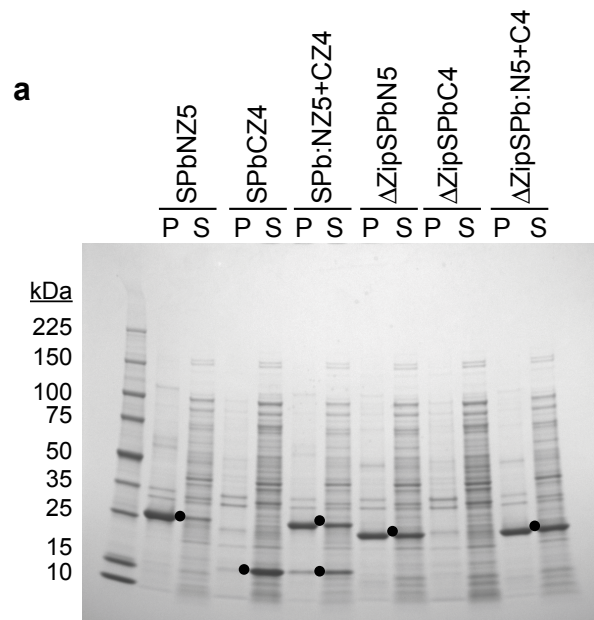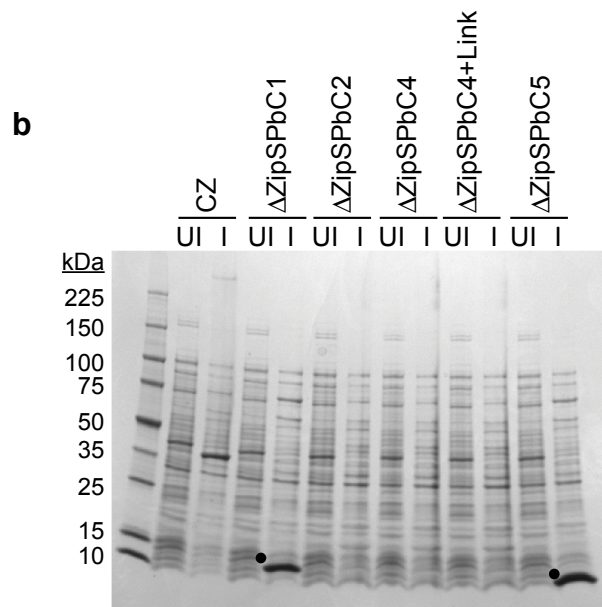

Supplement: S4 Fig — (a) Commassie stained SDS-PAGE gels showing insoluble (P) and soluble (S) fractions of SPbNZ5 and SPbCZ4, alone or coexpressed with or without leucine zippers. (b) Expression of the C-terminal fragments of LucY without leucine zipper, uninduced (UI) and induced (I). Bands of interest are indicated by a dot. (PDF) [file pone.0124272.s004.pdf]

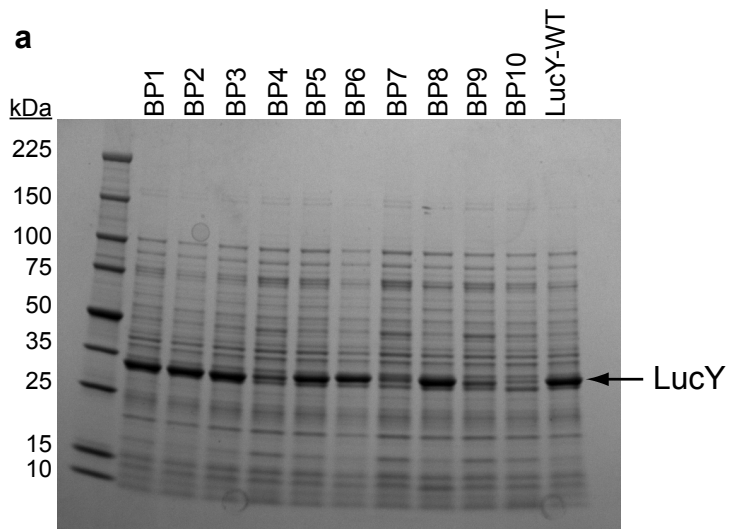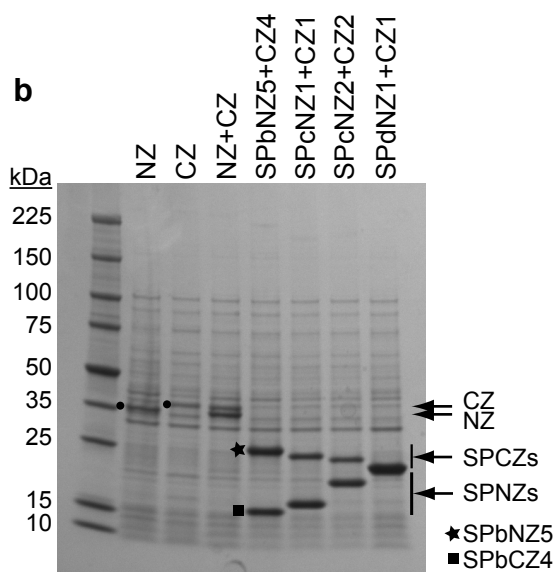

Supplement: S5 Fig — Commassie stained SDS-PAGE gels showing expression of (a) each circular permutation, designated BP1-10, and (b) split point trials derived from circularly permuted LucY. Corresponds to data in Fig 6a and 6b. (PDF) [file pone.0124272.s005.pdf]
