# Supplementary material for: LucY: A Versatile New Fluorescent Reporter Protein
Source: PLoS One. 2015 Apr 23;10(4):e0124272. doi: 10.1371/journal.pone.0124272 (PMC4408115; doi:10.1371/journal.pone.0124272)
Supplement: S1 Table — NZ constructs start with a 6x-His tag. CZ constructs end with the linker sequence SLSTPPTPSTPPT, followed by an Avi-tag. Split Pairs c and d were constructed from a circular permutation construct. (DOCX) [file pone.0124272.s006.docx]

**Supplemental Table 1.** Start and end residues for each split point (SP). NZ constructs start with a 6x-His tag. CZ constructs end with the linker sequence SLSTPPTPSTPPT, followed by an Avi-tag. Split Pairs c and d were constructed from a circular permutation construct.

|  | **Start** | **End** | **includes domains:** |
| --- | --- | --- | --- |
| **SPaNZ1** | D2 | G84 | 1 |
| **SPaNZ2** | D2 | A85 | 1 |
| **SPaNZ3** | D2 | G86 | 1 |
| **SPaNZ4** | D2 | L87 | 1 |
| **SPaNZ5** | D2 | D88 | 1 |
| **SPaCZ1** | A85 | R303 | 2&3 |
| **SPaCZ2** | G86 | R303 | 2&3 |
| **SPaCZ3** | L87 | R303 | 2&3 |
| **SPaCZ4** | D88 | R303 | 2&3 |
| **SPaCZ5** | H89 | R303 | 2&3 |
| **SPbNZ1** | D2 | P217 | 1&2 |
| **SPbNZ2** | D2 | P221 | 1&2 |
| **SPbNZ3** | D2 | S225 | 1&2 |
| **SPbNZ4** | D2 | N229 | 1&2 |
| **SPbNZ5** | D2 | H234 | 1&2 |
| **SPbCZ1** | V218 | R303 | 3 |
| **SPbCZ2** | C222 | R303 | 3 |
| **SPbCZ3** | I226 | R303 | 3 |
| **SPbCZ4** | P230 | R303 | 3 |
| **SPbCZ5** | A235 | R303 | 3 |
| **SPcNZ1** | L87 | P217 | 2 |
| **SPcCZ1** | V218 | G86 | 3&1 |
| **SPcNZ2** | L87 | H234 | 2 |
| **SPcCZ2** | A235 | G86 | 3&1 |
| **SPdNZ1** | P217 | G86 | 3&1 |
| **SPdCZ1** | L87 | Q216 | 2 |
