# Supplementary material for: LucY: A Versatile New Fluorescent Reporter Protein
Source: PLoS One. 2015 Apr 23;10(4):e0124272. doi: 10.1371/journal.pone.0124272 (PMC4408115; doi:10.1371/journal.pone.0124272)
Supplement: S2 Table — Amino acid numbering is that of wild-type. (DOCX) [file pone.0124272.s007.docx]

**Supplemental Table 2.** N- and C-terminal residues of LucY circular permutations, with order of domain occurrence listed. Amino acid numbering is that of wild-type.

|  | **N-term** | **C-term** | **Domain arrangement** |
| --- | --- | --- | --- |
| **BP1** | G84 | L83 | 2-3-1 |
| **BP2** | A85 | G84 | 2-3-1 |
| **BP3** | G86 | A85 | 2-3-1 |
| **BP4** | L87 | G86 | 2-3-1 |
| **BP5** | D88 | L87 | 2-3-1 |
| **BP6** | P217 | Q216 | 3-1-2 |
| **BP7** | P221 | N220 | 3-1-2 |
| **BP8** | S225 | G224 | 3-1-2 |
| **BP9** | N229 | R228 | 3-1-2 |
| **BP10** | H234 | D233 | 3-1-2 |
